# Using mixed methods and partnership to develop a program evaluation toolkit for organizations that provide physical activity programs for persons with disabilities

**DOI:** 10.1186/s40900-024-00618-7

**Published:** 2024-09-02

**Authors:** Sarah V. C. Lawrason, Pinder DaSilva, Emilie Michalovic, Amy Latimer-Cheung, Jennifer R. Tomasone, Shane Sweet, Tanya Forneris, Jennifer Leo, Matthew Greenwood, Janine Giles, Jane Arkell, Jackie Patatas, Nick Boyle, Nathan Adams, Kathleen A. Martin Ginis

**Affiliations:** 1 School of Health and Exercise Sciences, University of British Columbia, Kelowna, BC Canada; 2 International Collaboration on Repair Discoveries, Vancouver, BC Canada; 3 Abilities Centre, Whitby, ON Canada; 4 School of Kinesiology and Health Studies, Queen’s University, Kingston, ON Canada; 5 Revved Up, Kingston, ON Canada; 6 Department of Kinesiology and Physical Education, McGill University, Montreal, QC Canada; 7 Center for Interdisciplinary Research in Rehabilitation of Greater Montreal (CRIR), Montreal, Canada; 8 The Steadward Centre for Personal and Physical Achievement, University of Alberta, Edmonton, AB Canada; 9 Pickering Football Club, Pickering, ON Canada; 10 Rocky Mountain Adaptive, Canmore, AB Canada; 11 Active Living Alliance, Ottawa, ON Canada; 12 BC Wheelchair Sports Association, Vancouver, BC Canada; 13 Department of Medicine, University of British Columbia, Vancouver, BC Canada

**Keywords:** Implementation science, Knowledge translation, Delphi technique

## Abstract

**Background:**

The purpose of this paper is to report on the process for developing an online RE-AIM evaluation toolkit in partnership with organizations that provide physical activity programming for persons with disabilities.

**Methods:**

A community-university partnership was established and guided by an integrated knowledge translation approach. The four-step development process included: (1) identify, review, and select knowledge (literature review and two rounds of Delphi consensus-building), (2) adapt knowledge to local context (rating feasibility of outcomes and integration into online platform), (3) assess barriers and facilitators (think-aloud interviews), and (4) select, tailor, implement (collaborative dissemination plan).

**Results:**

Step 1: Fifteen RE-AIM papers relevant to community programming were identified during the literature review. Two rounds of Delphi refined indicators for the toolkit related to reach, effectiveness, adoption, implementation, and maintenance. Step 2: At least one measure was linked with each indicator. Ten research and community partners participated in assessing the feasibility of measures, resulting in a total of 85 measures. Step 3: Interviews resulted in several recommendations for the online platform and toolkit. Step 4: Project partners developed a dissemination plan, including an information package, webinars, and publications.

**Discussion:**

This project demonstrates that community and university partners can collaborate to develop a useful, evidence-informed evaluation resource for both audiences. We identified several strategies for partnership when creating a toolkit, including using a set of expectations, engaging research users from the outset, using consensus methods, recruiting users through networks, and mentorship of trainees. The toolkit can be found at et.cdpp.ca. Next steps include disseminating (e.g., through webinars, conferences) and evaluating the toolkit to improve its use for diverse contexts (e.g., universal PA programming).

**Supplementary Information:**

The online version contains supplementary material available at 10.1186/s40900-024-00618-7.

## Abstract (plain language summary)

Organizations that provide sport and exercise programming for people with disabilities need to evaluate their programs to understand what works, secure funding, and make improvements. However, these programs can be difficult to evaluate due to lack of evidence-informed tools, low capacity, and few resources (e.g., money, time). For this project, we aimed to close the evaluation gap by creating an online, evidence-informed toolkit that helps organizations evaluate physical activity programs for individuals with disabilities. The toolkit development process was guided by a community-university partnership and used a systematic four-step approach. Step one included reviewing the literature and building consensus among partners and potential users about indicators related to the success of community-based programs. Step two involved linking indicators with at least one measure for assessment. Step three involved interviews with partners who provided several recommendations for the online toolkit. Step four included the co-creation of a collaborative plan to distribute the toolkit for academic and non-academic audiences. Our comprehensive toolkit includes indicators for the reach, effectiveness, adoption, implementation, and maintenance of physical activity programs for individuals with disabilities. This paper provides a template for making toolkits in partnership with research users, offers strategies for community-university partnerships, and resulted in the co-creation of an evidence-informed evaluation resource to physical activity organizations. Users can find the toolkit at et.cdpp.ca.

**Supplementary Information:**

The online version contains supplementary material available at 10.1186/s40900-024-00618-7.

## Background

### Disability and physical activity

The United Nations Convention on the Rights of Persons with a Disability protects the rights of people living with disabilities to access full and effective participation in all aspects of life, including sports and other recreational forms of physical activity (PA) such as exercise and active play. But because of countless environmental, attitudinal and policy barriers [1], children, youth and adults with disabilities are the most physically inactive segment of society [2, 3]. Physical inactivity increases the risk that people with disabilities will experience physical and mental health conditions, social isolation, and stigma [4]. Systematic reviews have evaluated the effects of participation in PA programs among children, youth, and adults with physical, intellectual, mental, or sensory disabilities. Many, but not all, of these reviews have reported significant improvements in physical health, mental health, and social inclusion [2]. One reason for the inconsistent outcomes is that the PA participation experiences of people with disabilities are not universally positive [5].

Qualitative and quantitative research shows that people with disabilities often report negative PA experiences; for instance, being marginalized, excluded, and receiving sub-standard equipment, access, instruction, and opportunities to fully participate in PA [6–8]. Research and theorizing on quality PA participation and disability indicate that these low-quality PA experiences deter ongoing participation and undermine the potential physical and psychosocial benefits of PA for children and adults [5, 9]. These findings attest to the need for evaluation of existing PA programs to identify what is working, and where improvements are needed to achieve optimal participation and impact.

### Evaluating community-based programs

Persons with disabilities increasingly participate in disability sport to be physically active, and disability sport is often delivered by community organizations [2]. Like many community-based and non-profit organizations, organizations that provide PA programming for persons with disabilities (herein referred to as ‘this sector’) are often expected to conduct evaluations. These evaluations are done to secure and maintain external funding, demonstrate impact to board members and collaborators, and understand capacity for growth [10]. Even though program evaluations are often required, real-world programs are difficult to evaluate [11] and organizations often lack capacity and resources to conduct evaluations effectively [12]. Programs may be difficult to evaluate due to program complexity (e.g., setting, target population, intended outcomes; [11], and evaluation priorities (e.g., differing partner needs and resources; [13]. Organizations may lack capacity in understanding and using appropriate evaluation methods and tools [14], determining what counts as evidence and its application [15], and the roles of researchers and practitioners in supporting real-world program evaluations [16].

Evaluation frameworks can be used to facilitate a guided, systematic approach to evaluation. A framework involves an overview or structure with descriptive categories, meaning they focus on describing phenomena and how they fit into a set of categories rather than providing explanations of how something is working or not working [17]. One evaluation framework that is commonly applied in PA and disability settings is the RE-AIM framework [18]. RE-AIM is comprised of five evaluation dimensions or categories: (a) Reach: the number, proportion, and representativeness of individuals who engage in a program, (b) Effectiveness: the positive and negative outcomes derived from a program, (c) Adoption, the number, proportion, and representativeness of possible settings and staff participating in the program, (d) Implementation: the cost and extent to which the program was intended to be delivered, and (e) Maintenance: the assessment beyond six months at the individual and organizational levels. The RE-AIM framework is appropriate in this sector because it aligns with organizations’ need to understand factors that influence PA participation at both individual and organizational levels and for process (formative) and outcome (summative) evaluations [19–23]. Additionally, the RE-AIM framework has demonstrated feasibility to evaluate programs in this sector [19, 21, 22]. The RE-AIM framework was developed to address the failures and delays of getting scientific research evidence into practice and policy [18].

### Gaps between evaluation research and practice

There has been a growing body of evidence to suggest that one of the most effective ways to bridge the gap between research and practice is through integrated knowledge translation (IKT; [24]). IKT means that the right research users are meaningfully engaged at the right time throughout the research process [25]. IKT involves a paradigmatic shift from recognizing researchers as ‘experts’ to valuing the expertise of individuals with lived experience, programmers, and policymakers through their inclusion in the development of the research questions, methods, execution, and dissemination to ensure that the research is relevant, useful, and usable [25]. A commitment to IKT aligns with the “nothing about us without us” philosophy of the disability rights movement [26] and is therefore ideal for a toolkit development process for this sector.

To address the gaps of lack of evidence-informed resources and reduced organizational capacity to conduct program evaluations [12], our community partners (leaders from seven Canadian organizations in this sector) identified that a toolkit is needed. An evaluation toolkit is a collection of tools that includes materials that may be used individually or collectively, such as educational material, timelines, and assessment tools, and the tools may often be customized based on context, thus helping to bridge the translation gap between evidence and practice [27]. Toolkit development can be a multi-step process including literature reviews, interviewing partners, and using a Delphi approach [27]. Previous research with community-based disability PA organizations suggests that digital platforms can be an efficient way for participants and staff to provide evaluation access to evaluation tools [19, 23]. Together, this research culminated in our decision to (1) use RE-AIM for the toolkit’s framework, meaning the toolkit was organized using the five evaluation dimensions, and (2) to deliver the toolkit through interactive technology. The purpose of this paper is to report on a systematic, IKT-focused process for the design, development, and formulation of implementation considerations for an online RE-AIM evaluation toolkit for organizations that provide PA programming for persons with disabilities.

## Methods

### Research approach

A community-university partnership was established between seven Canadian disability PA organizations and three universities. A technology partner guided the back-end development of the online toolkit. Using an IKT approach [25], community partners were engaged before the research grant was written and submitted to ensure that the project was meaningful and focused on the appropriate tasks and outcomes. To guide our partnership, we agreed to adopt the IKT guiding principles for SCI research [25] which aim to provide a foundation for meaningful engagement between partners. An example of a guiding principle is partners share in decision-making [25]. The principles were presented at each bi-monthly team meeting and participants had the opportunity to share concerns if certain principles were not upheld. Partners had regular opportunities for sharing in decision making, provided financial contributions to accelerate the project, and benefitted from developing the toolkit to tailor indicators and measures relevant for disability PA organizations. Two community partner leaders also provided mentorship to academic trainees on community engagement in research, employment in non-academia, and project management, emphasizing the multi-directional nature of the partnership. To see the entire IKT process, see Appendix A in the supplemental file.

**Fig. 1:**
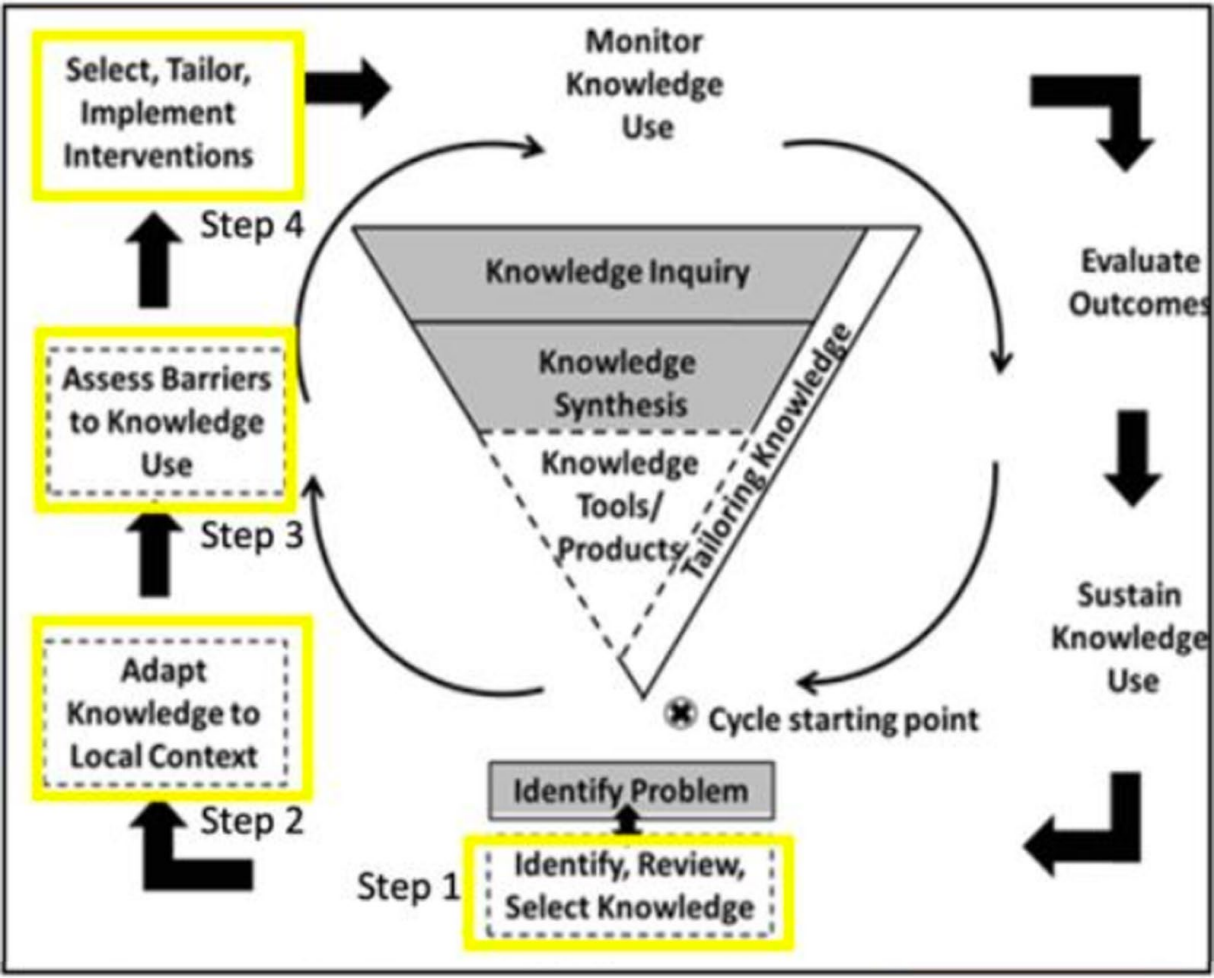
Knowledge to action framework (adapted from [28])

To maximize the likelihood that our toolkit is used in practice, our development process was guided by the Knowledge-to-Action (KTA) framework (see Fig. 1; [28]). The KTA framework was developed to help researchers with knowledge translation by identifying the steps in moving knowledge into action [28]. The KTA framework has two components: (a) knowledge creation and (b) action cycle. Our toolkit development process followed the steps of the action cycle, whereby existing knowledge is synthesized, applied, and mobilized. The problem to be addressed is a need for a program evaluation toolkit. To solve the problem, as shown with the yellow boxes in Fig. 1, the steps for developing the RE-AIM evaluation toolkit included: (1) identify, review, and select knowledge; (2) adapt the knowledge to the local context and users; (3) assess the barriers and facilitators to knowledge use; and (4) select, tailor, and implement the toolkit.

To guide toolkit development, we ensured the methods aligned with recommendations from the COnsensus-based Standards for the selection of health Measurement Instruments/ Core Outcome Measures in Effectiveness Trials (COSMIN/COMET) groups for generating a set of core outcomes to be included in health intervention studies [29]. These guidelines state that developing a core outcome set requires finding existing outcome measurement instruments (see Step 1), quality assessment of instruments (see Step 2), and a consensus procedure to agree on the core outcome set (see Step 2) [29].

### Step 1: Identify, review, and select knowledge

#### Literature review

The first step in identifying, reviewing, and selecting knowledge was to conduct a literature review. The literature review examined research using the RE-AIM framework to evaluate community-based and health-related programs. This was completed through a search of www.re-aim.org (which lists all RE-AIM evaluations) to identify indicators for each RE-AIM dimension within community-based and health-related contexts. Studies were included if they: used the RE-AIM framework to evaluate a community-based health program or involved persons with disabilities, were published in English, and were peer reviewed. All study designs were included. The review also examined qualitative and quantitative studies of outcomes of community-based PA programs for people with disabilities (e.g., [9]) and outcomes our own partners have used in their own program evaluations. These papers and outcomes were discussed and chosen during early partnership meetings to initiate a list of indicators. Examples of community-based programs included peer support programs for individuals with spinal cord injuries in Quebec. Data extracted from papers included indicators (and their definitions) and associated measures used for evaluations.

#### Delphi process

The second part in identifying, reviewing, and selecting knowledge involves critically appraising the relevant literature identified, to determine its usefulness and validity for addressing the problem [28]. To determine usefulness and validity, a consensus-building outreach activity was used—an online Delphi method. Briefly, the Delphi method is used to arrive at a group decision by surveying a panel of experts [30, 31]. The experts each respond to several rounds of surveys. Survey responses are synthesized and shared with the group after each round. The experts can adjust their responses in the next round based on their interpretations of the “group response.” The final response is considered a true consensus of the group’s opinion [30, 31]. Delphi was ideal for our partnership approach because it eliminates power dynamics from the consensus-building process and ensures every expert’s opinion is heard and equally valued. Previous research has demonstrated the utility of Delphi methods to generate consensus among disability organizations regarding the most important outcomes to measure in a peer-support program evaluation tool [32].

Delphi methodologies are considered a reliable means for achieving consensus when a minimum of six experts are included [33]. Therefore, we aimed to recruit a minimum of six participants from each target group (i.e., members of disability PA organizations and researchers). Partners were encouraged to invite members who may qualify and be interested in completing the Delphi process. Participants completed a two-round Delphi process and were asked to rate each RE-AIM indicator on a scale of 1 (not at all important) to 10 (one of the most important). An indicator was included if at least 70% of participants agreed it was “very important” (8 or above) [31]. Indicators that did not meet these criteria were removed from the list.

Retained indicators were then paired with at least one possible measure of that indicator (e.g., the ‘Positive Youth Development’ indicator was paired with the Out-of-School Time Observation instrument [34]). The partnership’s goal was to develop a toolkit comprised of valid and reliable measures. Therefore, the validity and reliability of each measure were critically appraised by the academic team-members using COSMIN/COMET criteria [29]. For some ‘Effectiveness’ indicators, published questionnaires were identified from the scientific literature. Measures were retained if they had high quality evidence of good content validity and internal consistency reliability [29] and were used in PA contexts and/or contexts involving participants with disabilities. The measures of all other indicators (where no published questionnaire measure was identified) were assessed by nine partners and modified to ensure that the measure was accurate and reliable for evaluation use in this sector.

### Step 2: Adapt knowledge to local context

In the KTA framework, this phase involves groups making decisions about the value, usefulness, and appropriateness of knowledge for their settings and circumstances and customizing the knowledge to their particular situation [28]. Using Microsoft Excel, partners were sent a list of the selected indicators and measures in two phases (Phase 1: “RE” indicators and Phase 2: “AIM” indicators). Partners were asked to rate, on a scale of 0 to 2 the following categories for each measure: feasibility-time (not at all feasible to feasible), feasibility-complexity (not at all feasible to feasible), accuracy (not at all accurate to accurate), and unintended consequences (no, maybe, yes). They were also asked to provide additional feedback. This step only involved partners on the project with experience administering questionnaires (in research or evaluation settings) because the process required knowledge of how to administer measures to respondents. The median and mean of each category were calculated with community partner responses given double weighting/value relative to academic partner responses. Double weighting was given to community partner responses as the toolkit is anticipated to be used more frequently in community settings. The feedback was summarized. Results were presented to all partners during an online meeting, and team members discussed feedback to establish agreement on measures. The measures were sent out to partners again to provide any final feedback on included indicators and measures. The selected indicators and measures were compiled in an online program evaluation toolkit compliant with accessibility standards.

### Step 3: Assess barriers and facilitators

In the KTA framework, this step involves identifying potential barriers that may limit knowledge uptake and supports or facilitators that can be leveraged to enhance uptake [28]. In Step 3, partners were invited to participate in an unstructured, think-aloud interview while they used the online program evaluation toolkit [35]. Interviews were conducted to collect detailed data about how users reacted to different parts of the toolkit content, format, and structure. Each interview was conducted over Zoom with one participant and two interviewers. The two-to-one interview format [36] supported the ability to take notes during the interview, ask questions from different perspectives, and reflect on common experiences to the two interviewers [36] with the website. Participants were also asked how the toolkit was used and any barriers to its use, and identified features of the toolkit that may need to be changed. In a separate group meeting, team members were asked for ideas on how to overcome potential barriers to using the toolkit and tips for its implementation. Data were analyzed using a content analysis approach [37] and recommendations were prioritized by the lead and senior authors using the MoSCoW method [38]. The MoSCoW method is a prioritization technique that has authors categorize recommendations using the following criteria: (a) “Must Have” (Mo), (b) “Should Have” (S), (c) “Could Have” (Co), and (d) “Won't Have This Time” (W). These recommendations were presented to all partners for further discussion. Based on the feedback, the toolkit content and technology were further iterated as needed. Information from this step was used to write brief user guides for toolkit users.

### Step 4: Select, tailor, implement

In the KTA framework, this step involves planning and executing interventions to promote awareness and implementation of knowledge, and tailoring interventions to barriers and audiences [28]. In Step 4, during an online partnership meeting, a brainstorming activity was completed to discuss target audiences for the toolkit, barriers and facilitators to outreach, and dissemination ideas. Team members formulated a dissemination plan and identified promotional resources they need to tailor the dissemination of the toolkit to their sector networks.

## Results

### Step 1: Identify, review, and select knowledge

#### Literature Review

The initial searching process on the re-aim.org database identified 15 papers with relevant indicators for a RE-AIM toolkit. These papers and their citations are in Appendix B in the supplemental file. Additional resources identified by partners included: [2, 9, 39, 40], and partners’ previous experiences with evaluations to inform potential indicator choices. In total, 62 indicators were identified across all RE-AIM domains.

#### Delphi process

**Fig. 2:**
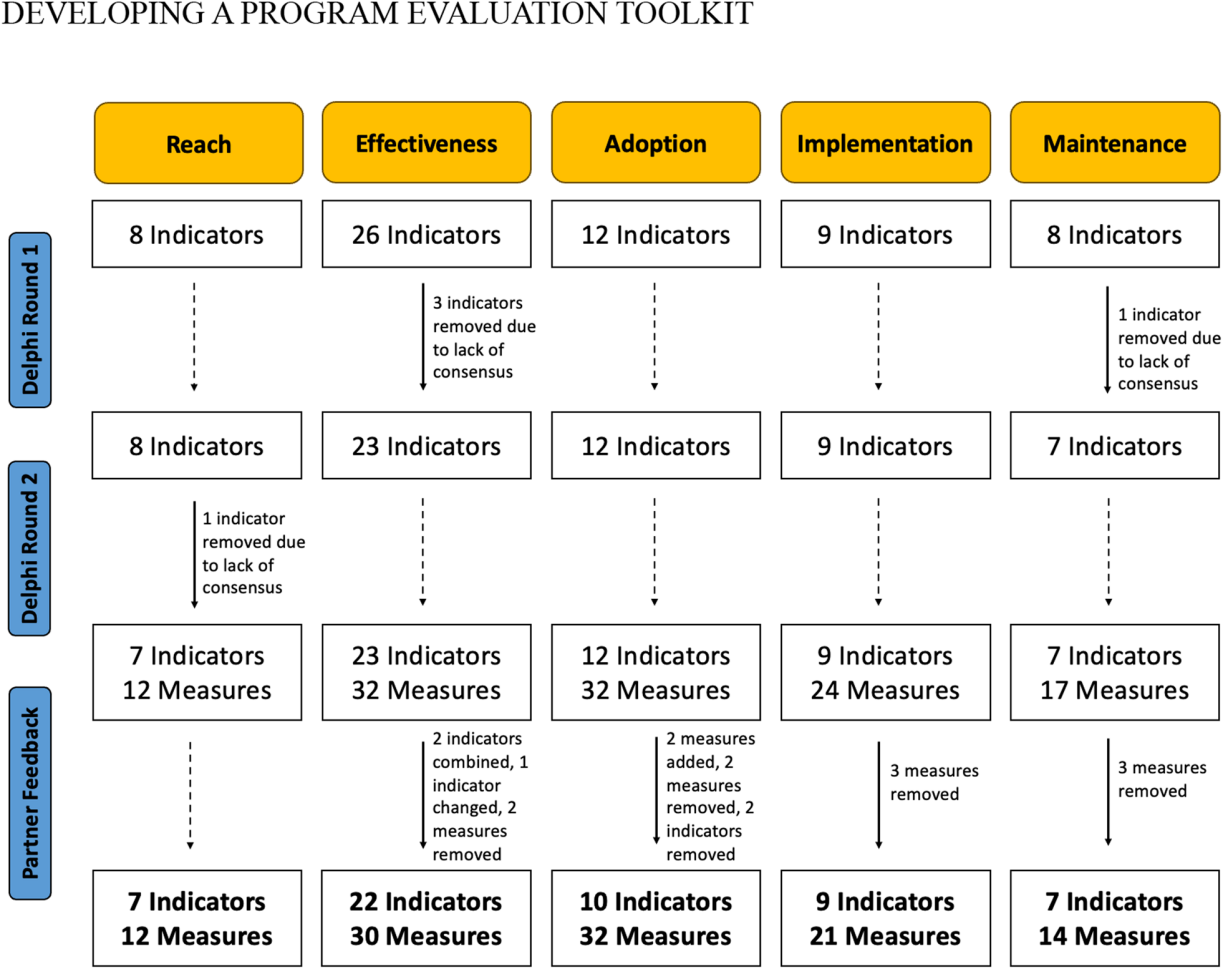
Adaptation process for indicators and measures from the Delphi process and partner feedback during COSMIN/COMET rating

In round 1, 32 people participated in the exercise (two participants did not provide demographic information). In round 2, 28 people completed the questionnaire (four participants did not provide demographic information). Detailed participant demographics are presented in Table 1. The adaptation of indicators through the Delphi process can be found in Fig. 2. Given that nearly all indicators were deemed important from round 2, we agreed that a third round of the Delphi process was not needed. Based on the literature review, measures for each indicator were identified.

**Table 1:** Demographic details for Round 1 and Round 2 Delphi participants

Characteristics	Round 1 (*n* = 32)	Round 2 (*n* = 28)
*Role (n)*		
Researcher/professor	7	5
Coach, Coach Developer, or Athlete	4	1
Executive Director	4	3
Program Manager, Coordinator, or Evaluator	12	9
Physical Activity Consultant, Provider, or Therapist	3	6
*Primary activity of organization (n)*		
Post-secondary institution	7	5
Program/service delivery	20	15
Program/service planning or evaluation	3	4
Years of experience with organization, Mean ± SD	4.70 ± 5.88	3.04 ± 2.60
*Province (n)*		
Alberta	7	5
British Columbia	12	5
Maritimes and Quebec	2	2
Ontario	9	12
Level of organization impact (%)*		
Local	80	71
Provincial	60	54
Federal	13	21
Types of disabilities served (%)*		
Physical disabilities	100	100
Intellectual disabilities	67	63
Sensory disabilities	57	67
*Individual attributes (%)*		
Age (years), Mean ± SD	35.27 ± 9.43	34.54 ± 8.31
Women	83	88
Identify as person with disability	10	0
Have family member with disability	20	21
Caregiver for person with disability	17	21

*Participants can select more than one answer

### Step 2: Adapt knowledge to local context

Eight partners (*n* = 3 academic, *n* = 5 community) completed the rating process for the “RE” domains and 10 partners (*n* = 3 academic, *n* = 7 community) completed the rating process for the “AIM” domains (rating feasibility, complexity, accuracy, and unintended consequences; see Table 2). Respondent feedback was used to adapt and improve the measures to make them more feasible, less complex, and more accurate to reflect the indicators properly. Respondents also suggested that each measure should also include information boxes about the respondents, administrators, type of data collection, and time to complete data collection. The adaptation of indicators and measures from this process can be found in Fig. 2. The final list of indicators and measures can be found in Table 3.

**Table 2:** Percentage of indicators with median ratings indicating feasibility (time, complexity), perceived accuracy, and no unintended consequences for RE-AIM measures

Domain	Number of measures assessed	Feasibility (time)	Feasibility (complexity)	Perceived accuracy	Unintended consequences
Reach	12	75	93	100	100
Effectiveness	32	91	94	100	100
Adoption	32	91	81	91	97
Implementation	23	96	100	100	100
Maintenance	17	71	88	76	100

**Table 3:** Indicators and their definitions and measures included in online toolkit (et.cdpp.ca)

Domain	Indicator	Definition	Measure(s)	Type of data collection
Reach	Target population	The individuals the program is trying to reach, or the individuals who are the target of the evaluation	Who is our target population? (e.g., athletes, coaches, volunteers)	Interviews/ Debriefs, Document Review
	Description of target population	Characteristics of the target population to describe the group	What are some specific characteristics of our target population? [Example response options: demographics (e.g., age, gender, ethnicity); disability-specific demographics (e.g., type, congenital vs. acquired, types of mobility aids); number of years in disability sport]	Document Review, Survey (Fixed)
	Community promotion	How the organization is promoting programs in the community	What kind of promotional material is [the organization] disseminating to reach the target audience? What types of events is [the organization] organizing to recruit or engage new participants?	Document Review, Interviews/ Debriefs
	Direct reach	How [the organization] is directly or physically reaching the target population	How many events has [the organization] hosted? How many individuals attended these events?	Document Review, Interviews/ Debriefs, Survey (Fixed)
	Awareness	Number of individuals aware of the organization or program. (Deliver this survey to the general population or target population)	Have you heard of (insert program or organization name)? If yes, how did you hear of (insert name)? [Provide response options: Email/Newsletter, social media, Word of mouth, etc.,]	Survey (Fixed), Interviews/ Debriefs
	Willingness to participate	The number of individuals in target population who are willing to participate in the organization or program	According to Twitter, Instagram and Facebook, how many comments from users’ express interest in participating in the program or organization? Based on resources sent out to prospective participants, how many individuals expressed interest by responding and who were they?	Website Analytics, Document Review
	Population need	Understanding the documented gaps in the organization for reach	After reviewing previous documentation, internal reports, and above evaluated reach measures, who is [the organization] not reaching? What is missing in [the organization's] efforts?	Document Review
Effectiveness	Positive and negative outcomes for members	The positive and negative outcomes that members have experienced from participating in the program /organization	What has been your experience with [insert program]? What impact has [insert program] had on your life in general? What are some benefits that you have gained from participating in [insert program]? Have you had any negative outcomes or disadvantages from participating in [insert program]?	Interviews/ Debriefs
	Member belief in effectiveness	The percent of members who believe the program/organization has helped them improve in terms of program/ organization goals	Do you feel that [insert program] has improved [insert program goal]? Why or why not?	Survey (Fixed), Survey (Open)
	Service access	The number of members who access other programs or services because of their membership in your organization/ program. Understanding how members access these services, and how these supports change over time	Have you accessed other programs or services because of your membership in [insert program]? How have you accessed these services? Have these supports changed over time?	Survey (Fixed), Interviews/ Debriefs, Survey (Open)
	Member satisfaction	How satisfied members are with the program/ organization	Are you satisfied with [insert program]? Why or why not? Do you have any recommendations on how the program can improve?	Survey (Fixed), Survey (Open), Interviews/ Debriefs
*Psychological outcomes*	Independence	One's ability to be self-sufficient	“I am more independent” (1-item)	Survey (Fixed)
	Life satisfaction	Feelings of satisfaction with one's own life, including self-satisfaction and satisfaction with different aspects of life	NIH Toolbox – General Life Satisfaction (5-items) (46)	Survey (Fixed)
	Meaning and purpose	Meaning in life refers to the feeling that one's life and experience make sense and matter. Purpose refers to the extent to which one experiences life as being directed, organized, and motivated by important goals	NIH Toolbox – Meaning and Purpose (7-items) [46]	Survey (Fixed)
	Confidence	Feeling self-assured about one's own qualities/ capabilities	1-item measure: "I am ______ that I can accomplish most things I set out to do." On a scale from: Much less confident, to Much more confident [47]	Survey (Fixed)
	Self-efficacy	A person's feelings of control over their life, as well as their confidence in being able to manage their own functioning and deal effectively with situations and demands	*Domain or task specific self-efficacy:* 1-item measure: “How confident are you in your ability to….” followed by specific skill: 10-point Likert scale anchored by not at all confident and extremely confident. [48] *General Self-Efficacy *(4-items) [49]	Survey (Fixed)
	Resilience	The ability to bounce back and recover from difficult/ stressful situations	Brief Resilience Scale (6-items) [50]	Survey (Fixed)
	Quality of Life	An individual's perceptions of their position in life and in relation to their goals, expectations, standards, and concerns. This concept incorporates a person’s physical health, psychological state, level of independence, social relationships, personal beliefs, and their relationships to salient features of the environment	WHO QoL BREF (26-items) [51]	Survey (Fixed)
*Social outcomes*	Social support	Perceived and received support that is intended to enhance the well-being and positive outcomes of the recipient	Social Support and Exercise Survey (13-items) [52]	Survey (Fixed)
	Sport and community inclusion	Making sure everybody has the same opportunities to participate in every aspect of sport and community to the best of their abilities and desires. Inclusion requires making sure that adequate policies and practices are in effect in a community or organization	Social Inclusion Measure (12-items) [53]	Survey (Fixed)
*Physical health*	Physical activity	Any bodily movement produced by skeletal muscles that requires energy expenditure. Physical activity refers to all movement including during leisure time, for transport to get to and from places, or as part of a person's work	International Physical Activity Survey: 7-items, estimates days/week, hours/day, and minutes/day of intensity of physical activity and sitting time [54]	Survey (Fixed)
	Health	The overall evaluation of one's physical and mental health	Global Health Scale (7 to 10-items) [55, 56]	Survey (Fixed)
	Physical self-concept	A combination of individual self-perceptions related to physical appearance, athletic abilities, and physical capacities	Physical Self-Description Survey Short Form (47-items) [57]	Survey (Fixed)
*Organizational outcomes*	Sport organization capacity	The overall capacity of an organization to produce the outputs and outcomes it desires. Partners will benefit from an oversight of the sport organization including: 1) The organization’s capacity to deliver on various projects and to find the right 'fit'. 2) It provides a measure of the sophistication of the organization	Organizational Capacity Variables (20-items) [58]	Survey (Fixed), Document Review
	Volunteer inspiration	Feeling of excitement and energy to take action to volunteer	Inspiration Scale (4-items) [59]	Survey (Fixed)
	Volunteer intention	The goal, reason, or purpose one has for volunteering	Intention to return to volunteering (5-items) [60]	Survey (Fixed)
*Program outcomes*	Quality participation	An athlete's broad subjective evaluation that their sport involvement is satisfying, enjoyable, and generates personally valued outcomes	Measure of Experiential Aspects of Participation (12-items) [61]	Survey (Fixed)
	Positive youth development	Extent to which youth participants enhance their skills, beliefs in the future, self-regulation, self-efficacy as well as social, emotional, cognitive, and behavioural competence	Out-of-School Time Observation Instrument (26-items) [34]	Observation (Fixed)
	Program quality	Refers to the structures and processes within a program that relate to participant outcomes. Program structures refer to an organization's capacity to deliver a program to youth (e.g., physical space, staffing, funding, community collaborations). Program processes refer to how the program is delivered (e.g., supportive relationships, opportunities for skill-building, autonomy)	Program Quality Assessment in Youth Sport (51-items) [62]	Observation (Fixed)
Adoption	Member-Type Adoption	The number of people from the target population who are members in the program /organization, and how they are they involved (e.g., coach, volunteer, athlete)	How many members (according to member-type, e.g., athlete, staff) are in our program? How many people are estimated in your target population according to member type?	Document Review
	Engaged adoption	The total number of members that have become actively involved in a program/ initiative	How many members in your organization have adopted (or are actively participating in) a specific program/initiative?	Document Review
	Description of adopters	Demographics/description of members and staff who have adopted the program. This should be carefully considered about whether the information needs to be collected or not	10 Questions, Ex: What is your age? [Provide response options] What is your gender? [response options provided]	Survey (Fixed and Open)
	Accessibility and ease of adoption	Assess program /organization access to information and evaluated delivery of the program	What resources (delivery method e.g., pamphlets, mail, email) and information (content e.g., program information) is sent formally to new participants?	Document Review
	Dropouts	Dropouts from program or organization. Number of former program or organization members	How many people were registered in your program at the beginning of a season/program year? How many participants stayed in the program until the end of a season? How many participants registered for the next season?	Document Review
	Attrition rate	The rate at which program members are no longer participating	Using your answers from “Dropouts”, what percentage of individuals dropped out of the program? (Divide the number of dropouts by the number of people registered in the program?)	Document Review
	Usability	Staff satisfaction with organization or program	In an overall, general sense, how satisfied are you with the [insert program]? [Response options scale, 1–5] Why or why not? Do you have any recommendations on how the program can improve? [Provide response options]	Survey (Fixed and Open); Interviews/ Debriefs
	Professional development and contributions	The number and type of professional development opportunities available for staff	How many opportunities for professional development (e.g., workshops) are offered to staff and/or volunteers by the organization? How many people are eligible (i.e., qualified to participate) for each professional development opportunity?	Document Review, Survey (Fixed and Open), Interviews/ Debriefs
	Demand for Tools	The specific tools, resources, and/ or philosophies required for staff to aid their ability to promote program adoption	What kind of resources/tools have helped you in your ability in promoting program participation? What kind of philosophies and skills do you hold that aid in program participation?	Survey (Open); Interviews/ Debriefs
	Support and Commitment of Staff and Management	Staff perceptions about the organization's commitment and coordination by management on facilitating program adoption	What is the organization's commitment to facilitate program participation? Do you think it is enough? Why or why not? What is the organization's coordination to facilitate program participation? Do you think it is enough? Why or why not?	Survey (Open); Interviews/ Debriefs
Implementation	Adherence and commitment	The extent to which program or organizational goals are applied in programs. Measuring how well the organization adheres to implementing the program	1. How well do you think [insert goal] is applied in [insert program]? Why or why not? 2. How well does the organization adhere to program implementation?	Survey (Open); Interviews/ Debriefs
	Compatibility	Evaluate how well the actual implementation of the program compares to the intended implementation and organizational guidelines for the program	1. Can you discuss how the written goals of your program (mission statements, curriculums, etc.) are targeted regularly through the programming? 2. Does your program include the appropriate components according to the Program Quality Assessment in Youth Sport?	Document Review; Interviews/ Debriefs [Resource intensive], Connected to 'Program Quality Assessment in Youth Sport' measure
	Program Cost and funding	The cost of resources needed to run the program /organization and the funding received to subsidize cost	1. Using financial documents, what is the cost of resources (e.g., staff, equipment; total cost) required by all initiatives and programs? 2. What is the annual budget for your program? [Try to answer this question when budget information is available] 3. What is the total income received for program? (e.g., sponsorships, grants, yearly fees)	Document Review
	Delivery	The skills used by program implementers in order to deliver the program or attain organizational goals	1. What do you think makes the program implementation successful or not successful? Why? What do you think can be improved? 2. What are some of your skills that you use when delivering the program?	Survey (Open); Interviews/ Debriefs
	Contact	The type and amount of contact the program/organization has with members. For instance, the number of direct contacts (e.g., phone calls, email, and personal communication) a program or organization has with members	1. How many phone contacts occur between the organization and members? 2. How many email contacts occur between the organization and members? [in a given time frame] 3. What other types of communication occur between the organization and members?	Document Review
	Support from management	Whether management supports the implementation of goals in programs	1. What support do you receive from management to implement the program? (e.g., time, resources, knowledge) 2. Do you think the support you receive is adequate? Why or why not? 3. How could management provide more support for program delivery?	Survey (Open); Interviews/ Debriefs
	Staff skills	The training provided to staff, coaches, and volunteers to implement program goals	1. What staff skills are fostered from staff engaging in training opportunities? Provide examples (leadership, professional coaching skills etc.)	Survey (Open); Interviews/ Debriefs
	Time expenditure	The amount of time spent implementing programs	1. What activities are involved in delivering [insert program] in [given time frame]? [Activities include lesson plans, implementing and planning program] 2. How much time do you spend on those activities?	Document Review; Interviews/ Debriefs
	Cost benefit analysis	Whether various staff tasks have a benefit for the overall program or organization. The extent to which staff costs (e.g., staff training, salaries, number of staff) provide program benefits (e.g., recruitment, adoption) or participant benefits (e.g., improved performance or quality of life)	1. What is the total cost of your program in [given time frame]? 2. Identify the most important positive outcomes for your program (adoption or see effectiveness measures, e.g., participation, quality of life). How many people experienced a positive change or received a certain score in that positive outcome? 3. What is the cost of each individual member who received the desired benefit listed in the previous question? (Divide total cost of program by number of members in relevant group, i.e., those who received benefit). Example: Total cost of program: $5,000. Benefit = participation; number of members: 50. Benefit = gaining independence; number of members who improved independence = 30. Cost–benefit for participation: $5,000/50 members = $100 per member. Cost–benefit for gaining independence: $5,000/30 members = $166.67	Document Review
Maintenance	Continuity of members	Average number of years individuals have been members of program/ organization	What is the average number of years an individual is a member? (Total membership years divided by total members)	Document Review [May be difficult to collect this information]
*Individual*				
	Outcome assessment	Evaluating how implementation of program/ organization goals changes over time. Comparing the initial program implementation with the current program implementation in terms of outcomes	How has your ability to implement program tools for program goals changed and compared over time? How have 'Effectiveness' outcomes changed over time (e.g., season per season, year per year)?	Document Review; Interviews/ Debriefs
	Facilitators and Barriers	Members' experiences with the program and any factors that promote or inhibit their ability to participate	1. What are your experiences with participation in [insert program]? 2. What has enabled your ability to participate in [insert program] over time? [Response options provided—family, transportation, fee support] 3. What has hindered your ability to participate in [insert program] over time? [Response options provided—family, transportation, fee support]	Survey (Fixed and Open); Interviews/Debriefs
*Organizational*	Program Plan	Evaluate the program's/ organization's long-term plan for future success. For instance, understanding how the program will be maintained to achieve longevity in the community	1. Using documents, how have program plans been used to plan and achieve goals in the future? (e.g., 1–2 yrs, 2–5 yrs, 5–10 yrs) 2. How do you plan on maintaining the program within the community? What strategies will you use and why?	Interviews/ Debriefs, Document Review
	Cost	Evaluate the financial plan in place for maintaining program/ organizational costs. Understanding the financial sustainability of the program with respect to costs compared to income	1. Does the program receive ongoing funding? 2. Is the program based on a fee-for-service funding model to help offset the costs?	Document Review
	Capacity building	Evaluate staff training opportunities and the extent to which these opportunities improved staff confidence to implement programs, staff skills, or skills to work with members	1. How many staff training opportunities are there over time? 2. What staff skills arise from those training opportunities over time? 3. How many training opportunities on average achieve goal of changing staff skills? (Divide total number of times/sessions/years of training opportunity by number of times/sessions/years achieving staff skill goal)	Document Review
	Embeddedness in System	How the program /organization is incorporated with the rest of the sport system	1. Using documents to determine goals achieved, how do these programs achieve goals that are related to parent organizations? (e.g., PSOs, NSOs). 2. Using documents, how many partnerships does your program have with other organizations? 3. How well is your sport program embedded within an able-bodied sport/exercise/physical activity system? Why or why not?	Document Review [Can take time]; Survey (Fixed and Open)

### Step 3: Assess barriers and facilitators

Six partners (community and academic partners) participated in unstructured think-aloud interviews, one of which was conducted jointly with two partners (*M*_*time*_ = 43.37, *SD* ± 13.50 min). Across interviews, 45 unique recommendations were identified for improving the usability of the toolkit. These recommendations were sorted using the MoSCoW method, and prioritized based on budgetary constraints, team skillsets, and competing needs. Of the 45 recommendations, 30 were identified as ‘Must haves’, 6 as ‘Should haves’, 4 as ‘Could haves’, and 5 as ‘Won’t haves’ (see Appendix C in the supplemental file). All 30 ‘Must have’ recommendations were implemented in collaboration with the technology partner, along with 2 ‘Should have’ recommendations.

### Step 4: Select, tailor, implement

After all recommendations were executed by the technology partner, a final project meeting was held to discuss project updates, barriers and facilitators to outreach, and ideas for dissemination. Barriers to outreach included lack of research or evaluation knowledge to use the toolkit, lack of funding to conduct evaluations, poor turnover from reaching users (i.e., users becoming aware of the toolkit) to receiving (i.e., users browse the toolkit website) to using the toolkit (i.e., users use the toolkit for an evaluation), and challenges connecting with hard-to-reach organizations. Facilitators to outreach included providing resources for evaluation support, connecting with trainees to support evaluations, having positive self-efficacy and attitude for conducting evaluation, building awareness on the benefits of the toolkit through a dissemination campaign, credibility in the toolkit development process, and reaching out to key funders for administration of toolkit as guidance.

The toolkit can be found at et.cdpp.ca and is intended to be used by community organizations and academic institutions that conduct program evaluations involving PA and disability (and inclusive integrated programming). This interactive toolkit allows users to customize to their program evaluation situation by selecting a) which RE-AIM dimensions they want to evaluate, and b) which indicators they want to measure within a particular RE-AIM dimension (e.g., self-efficacy and quality participation within the Effectiveness dimension). Based on users’ selections, the toolkit program compiles the corresponding measures for each indicator into a customized, downloadable document that the user can then put in the format of their choosing (e.g., online survey, paper questionnaire) for their program evaluation. This design aligns with partner requests for a simple online interface that provides flexibility and tailoring to their program evaluation needs. The toolkit and user guides are made freely available (i.e., open access), to maximize accessibility to community organization and academic audiences.

A plan with dissemination and capacity building activities was created to ensure the supported uptake of the evaluation toolkit. Our priority was to create a knowledge translation and communications package (e.g., newsletter article, social media content) for community partner organizations to disseminate through their channels. This included disseminating information to other community organizations within their network and funding partners (e.g., Sport Canada, Canadian Tire Jumpstart, ParticipACTION, provincial ministries, and the Canadian Paralympic Committee). This package served as the official ‘launch’ of the evaluation toolkit on July 20, 2023. Through this package, other activities were listed as potential ‘services’ interested parties can use. These services include bookable time for ‘office hours’ whereby a one-on-one meeting on how to use the toolkit and conduct program evaluation can be arranged and a 1-h ‘frequently asked questions’ webinar/workshop. Other activities included publishing an open-access manuscript, writing knowledge translation and media blogs about the manuscript, and delivering academic and community conference presentations.

## Discussion

The purpose of this paper was to report on the process of developing an evaluation toolkit in partnership with organizations that provide PA programming for persons with disabilities. Informed by the RE-AIM framework [18] and the knowledge-to-action framework [28], the toolkit development process involved a literature review, Delphi process, and interviews to adapt indicators and measures. Recommendations from partners were implemented, and the final toolkit can be found at et.cdpp.ca. Partners collaborated to create a dissemination and capacity building plan to support the uptake of the toolkit across the target audience.

Community organizations struggle to conduct program evaluations and to use existing evaluation frameworks. A recent scoping review identified 71 frameworks used to evaluate PA and dietary change programs [41]. Despite access to many frameworks, Fynn et al. [41] found limited guidance and resources for using the frameworks. In response to these concerns, the toolkit acts as a resource for using the RE-AIM framework by facilitating the uptake of evidence-informed evaluation practices. The toolkit will help organizations overcome barriers to evaluation identified by previous research by increasing capacity to use appropriate methods and tools [14] and providing education on determining what counts as evidence and data [15]. This can facilitate better organizational direction, improved programming, and importantly, better quality PA experiences for individuals with disabilities. The toolkit also complied with accessibility standards, an important benchmark for our partnership and a necessary step when creating a product for organizations that serve persons with disabilities. Accessibility standards were relatively easy to achieve and should be customary in all IKT activities.

To the best of our ability, the toolkit was developed specifically for organizations that provide programming for people with disabilities by focussing the literature review, having program partners in the disability community participate in the Delphi process, and ensuring the validity and reliability of indicators in disability contexts. However, there is an enormous shortage of data related to PA and disability as most national health surveillance systems exclude or do not measure disability [2]. While this general limitation may affect the toolkit, it also means that the toolkit may be useful for universal PA organizations that are interested in evaluating programs with non-disabled individuals. Additional research is needed to examine the effectiveness of the toolkit in diverse contexts.

This project provides a template for developing open-access, online evidence-informed toolkits using an IKT approach with community partners. There are few resources on how to develop toolkits for the health and well-being field informed by knowledge translation frameworks or that include perspectives of end-users (e.g., [42, 43]). The four-step mixed-methods approach was guided by the systematic use of frameworks to inform toolkit development. Our project utilized a rigorous, step-by-step process for creating toolkits and resources for this sector that centres the knowledge and expertise of research users. To centre the knowledge and expertise of research users, we employed several strategies identified by Hoekstra et al. [44] for building strong disability research partnerships. Important strategies for partnership when developing a toolkit include (1) using a set of norms, rules, and expectations, (2) engagement of research users in the planning of research, (3) using consensus methods (i.e., Delphi), and (4) recruiting research users via professional or community networks [44].

First, we used the IKT Guiding Principles [25] as the set of norms, rules, and expectations to guide our partnership. These principles were addressed throughout the partnership and provided criteria to understand the success of the partnership. Second, we engaged with community partners from the beginning of the research process. Working with community partners who were committed to developing a high-quality product was integral to the success of this project. Community partners were committed and highly engaged as the toolkit stemmed from a community-identified need, rather than solely a ‘research gap’. Third, using consensus methods is an excellent strategy to avoid decision-making that is dominated by certain voices or interests in the partnership [45]. One way that our project allowed for multiple voices to be heard was through our anonymous Delphi processes, which encouraged partners to share their input in a non-confrontational and data-driven manner. Fourth, in our partnership, many individuals and organizations had longstanding working relationships and aligned priorities for the project. Building our partnership based on previous trusting, respectful relationships was essential and using the IKT guiding principles [25] ensured that we maintained similar values and priorities throughout the partnership.

We used an additional strategy that has not been previously mentioned in the IKT literature: mentorship of research trainees by community partners. Through monthly meetings, two community partners provided mentorship sessions to three trainees. These sessions focused on how to close the research-to-practice gap and helped to facilitate strong relationships between researchers and research users. Mentorship was an important step for training the next generation of researchers to use IKT.

### Limitations

This project has some limitations. First, an exhaustive systematic scoping review was not conducted to identify evaluation indicators. This may have limited the number of relevant evaluation indicators included in the Delphi surveys. However, given that only five indicators were removed, and none were added after two rounds of Delphi, we are confident that our search returned relevant indicators. In the future, it may be worthwhile to consider an in-person or video-conference-facilitated Delphi process to encourage discussion and differentiation of indicators. Second, we identified several barriers and facilitators for using the toolkit, but addressing these barriers meaningfully was beyond the scope of this paper. We are currently in the process of disseminating (e.g., social media campaigns, blogs, discussions with funders) and evaluating the toolkit (e.g., surveys, using data analytics). This data will be reported in a future paper. Third, the interviews revealed 45 unique recommendations for the website and toolkit, but only some of these recommendations could be implemented due to budgetary constraints (e.g., adding a search function and filtering indicators to the website could not be completed).

## Conclusions

In summary, this paper reports on the development of an online, open-access program evaluation toolkit for the disability and PA sector. The toolkit is informed by the RE-AIM framework [18] and available at et.cdpp.ca. Our paper describes a four-step process guided by the KTA framework [28] and IKT principles [25] to work with community partners to ensure the toolkit is relevant, useful, and usable. The process included reviewing the literature, building consensus through two rounds of Delphi surveys, rating the feasibility and complexity of measures, assessing barriers and facilitators through think-aloud interviews, and crafting a dissemination and capacity-building plan. This paper provides a template for creating toolkits in partnership with research users, demonstrates strategies to enable successful community-university partnerships, and offers an evidence-informed evaluation resource to organizations that provide PA programming for persons with disabilities.

### Supplementary Information

**Additional file 1**.

**Additional file 2**.

## Data Availability

The datasets used and/or analysed during the current study are available from the corresponding author on reasonable request.

## Abbreviations

IKT: Integrated knowledge translation
KTA: Knowledge-to-action
PA: Physical activity
RE-AIM: Reach, effectiveness, adoption, implementation, maintenance
