# Supplementary material for: Using mixed methods and partnership to develop a program evaluation toolkit for organizations that provide physical activity programs for persons with disabilities
Source: Res Involv Engagem. 2024 Sep 2;10:91. doi: 10.1186/s40900-024-00618-7 (PMC11367884; doi:10.1186/s40900-024-00618-7)
Supplement: Supplementary file 2 — Additional file 2. [file 40900_2024_618_MOESM2_ESM.docx]

**Supplemental File**

**Appendix A:** Integrated Knowledge Translation Process

| **Research Stage** | **Type of Activity** | **Activity Description** |
| --- | --- | --- |
| **Conceptual design (grant)** | Discussion; Writing grant | The research question and methodology were co-developed by partners all partners prior to writing a funding grant. Community and academic partners (SVCL, PDS, EM, ALC, JRT, SS, TF, JL, KMG) contributed towards writing sections of the grant. |
| **Recruitment** | Developing and distributing recruitment materials (emails) | SVCL and KMG created recruitment materials (emails) to send to potential participants for the Delphi study.  PDS, EM, ALC, JRT, SS, TF, JL, MG, JG, JA, JP, NB reviewed and approved recruitment materials, sent emails to potential participants, and coordinated their participation with the study lead (SVCL) if necessary. |
| **Data collection and analysis** | Delphi process | All partners discussed, through an online meeting, the best methods and format for conducting the Delphi online. SVCL and a research assistant set up all Delphi materials in Qualtrics. All partners either participated in the Delphi or sent the surveys to their networks.  SS provided methodological input on survey cut-points. SVCL and a research assistant analyzed the data. All partners reviewed the data in a meeting to agree on next steps. |
|  | Excel COSMIN/ COMET rating of indicators and measures | All partners discussed, through an online meeting, the best methods and format for conducting the rating of indicators. NA and SVCL set up the indicators in the Excel sheets. PDS, EM, JRT, SS, JG, and JL participated in the rating process. NA and SVCL analyzed the data and discussed with all partners in an online meeting. All partners made additional changes and came to consensus on final indicators and measures. |
|  | Interviews | JRT and KMG helped to recruit additional partners for interviews. SVCL and NA conducted interviews. PDS, EM, JG, JL participated in interviews. SVCL and KMG analyzed data. Data was brought to all partners in an online meeting to discuss feasible recommendations to implement. |
| **Manuscript preparation** | Email | SVCL led the writing of the manuscript. KMG provided initial and final reviews. NA prepared a figure for the manuscript. All other authors reviewed the manuscript and provided feedback before submission for publication. |

**Appendix B:** Relevant papers identified to inform potential RE-AIM indicators.

| **Literature included in review** |
| --- |
| (Evans et al., 2015)  (Finch & Donaldson, 2010)  (Finch et al., 2011)  (Glasgow et al., 2006)  (Hone et al., 2015)  (Kohn et al., 2015)  (Koorts & Gillison, 2015)  (Lai et al., 2019)  (Lawrason et al., 2021)  (Schaap et al., 2018)  (Schwingel et al., 2016)  (Shirazipour et al., 2019)  (Shirazipour & Latimer-Cheung, 2020)  (Sweet et al., 2014)  (Williams et al., 2017) |

Evans, M. B., McGuckin, M., Gainforth, H. L., Bruner, M. W., & Côté, J. (2015). Coach development programmes to improve interpersonal coach behaviours: a systematic review using the re-aim framework. *British Journal of Sports Medicine*, *49*(13), 871. https://doi.org/10.1136/bjsports-2015-094634

Finch, C. F., & Donaldson, A. (2010). A sports setting matrix for understanding the implementation context for community sport. *British Journal of Sports Medicine*, *44*(13), 973. https://doi.org/10.1136/bjsm.2008.056069

Finch, C. F., Gabbe, B. J., Lloyd, D. G., Cook, J., Young, W., Nicholson, M., Seward, H., Donaldson, A., & Doyle, T. L. A. (2011). Towards a national sports safety strategy: addressing facilitators and barriers towards safety guideline uptake. *Injury Prevention*, *17*(3), 1. https://doi.org/10.1136/ip.2010.031385

Glasgow, R. E., Nelson, C. C., Strycker, L. A., & King, D. K. (2006). Using RE-AIM Metrics to Evaluate Diabetes Self-Management Support Interventions. *American Journal of Preventive Medicine*, *30*(1), 67–73. https://doi.org/https://doi.org/10.1016/j.amepre.2005.08.037

Hone, L. C., Jarden, A., & Schofield, G. M. (2015). An evaluation of positive psychology intervention effectiveness trials using the re-aim framework: A practice-friendly review. *The Journal of Positive Psychology*, *10*(4), 303–322. https://doi.org/10.1080/17439760.2014.965267

Kohn, M., Belza, B., Petrescu-Prahova, M., & Miyawaki, C. E. (2015). Beyond Strength: Participant Perspectives on the Benefits of an Older Adult Exercise Program. *Health Education & Behavior*, *43*(3), 305–312. https://doi.org/10.1177/1090198115599985

Koorts, H., & Gillison, F. (2015). Mixed method evaluation of a community-based physical activity program using the RE-AIM framework: Practical application in a real-world setting. *BMC Public Health*, *15*(1), 1102. https://doi.org/10.1186/s12889-015-2466-y

Lai, J., Klag, M., & Shikako‐Thomas, K. (2019). Designing a program evaluation for a medical‐dental service for adults with autism and intellectual disabilities using the RE‐AIM framework. *Learning Health Systems*, e10192. https://doi.org/10.1002/lrh2.10192

Lawrason, S., Turnnidge, J., Tomasone, J., Allan, V., Côté, J., Dawson, K., & Martin, L. J. (2021). Employing the RE-AIM Framework to Evaluate Multisport Service Organization Initiatives. *Journal of Sport Psychology in Action*, *12*(2), 87–100. https://doi.org/10.1080/21520704.2020.1773592

Schaap, F. D., Dijkstra, G. J., Finnema, E. J., & Reijneveld, S. A. (2018). The first use of dementia care mapping in the care for older people with intellectual disability: a process analysis according to the RE-AIM framework. *Aging & Mental Health*, *22*(7), 912–919. https://doi.org/10.1080/13607863.2017.1401582

Schwingel, A., Gálvez, P., Linares, D., & Sebastião, E. (2016). Using a Mixed-Methods RE-AIM Framework to Evaluate Community Health Programs for Older Latinas. *Journal of Aging and Health*, *29*(4), 551–593. https://doi.org/10.1177/0898264316641075

Shirazipour, C. H., & Latimer-Cheung, A. E. (2020). Understanding quality participation: exploring ideal physical activity outcomes for military veterans with a physical disability. *Qualitative Research in Sport, Exercise and Health*, *12*(4), 563–578. https://doi.org/10.1080/2159676X.2019.1645037

Shirazipour, C. H., Tennant, E. M., Aiken, A. B., & Latimer-Cheung, A. E. (2019). Psychosocial Aspects of Physical Activity Participation for Military Personnel with Illness and Injury: A Scoping Review. *Military Behavioral Health*, *7*(4), 459–476. https://doi.org/10.1080/21635781.2019.1611508

Sweet, S. N., Ginis, K. A. M., Estabrooks, P. A., & Latimer-Cheung, A. E. (2014). Operationalizing the RE-AIM framework to evaluate the impact of multi-sector partnerships. *Implementation Science*, *9*(1), 74. https://doi.org/10.1186/1748-5908-9-74

Williams, T. L., Ma, J. K., & Martin Ginis, K. A. (2017). Participant experiences and perceptions of physical activity-enhancing interventions for people with physical impairments and mobility limitations: a meta-synthesis of qualitative research evidence. *Health Psychology Review*, *11*(2), 179–196. https://doi.org/10.1080/17437199.2017.1299027

**Appendix C: Recommendations from partner interviews**

|  | Must-have | Should-have | Could-have | Won’t-have |
| --- | --- | --- | --- | --- |
| ‘About Us’ Page | - Tell people about the process of developing toolkit (4): Demonstrate credibility for selecting measures and evaluating tools, helpful for people to trust toolkit (4) - Add an ‘About Us’ page (3) |  |  |  |
| ‘Instructions/ Guidance’ Page | - Make guidance page language more ‘lay’ and less ‘research’ (4) - Encourage programs to identify specific outcomes and make this more visible on guidance page (2) - Clarify who the toolkit target audience is in the guidance page (2) - Having people think about what funders want reported (e.g., impact and summative outcomes) (1) - Provide examples of types of evaluation designs (e.g., pre-post survey, observation, post-survey) (1) - May need to tell users to create ID codes if using repeated measures (1) - Include statement be cautious about conclusions (other factors may be influencing outcomes that are not just the program itself but also impact the overall “success” of the program) (1) - Need to uphold administrative fidelity and consider administrative capacity (1) - Include a statement about ethics and marginalized groups (e.g., Indigenous community) - Have your evaluation framework in place before going to the build page (1) - Provide examples of how to use toolkit and goals for evaluation (1) - Add definitions/glossary page e.g., program, outcomes (1) - Highlight and reiterate data that program already collects and capacity to collect more data (1) | - Incorporate other resources and add a resources tab (4)* - Allow people to save progress (2) - Ask questions to guide people through guidance page: (1) Do you have your target population? Do you have your evaluation goals? | - Trigger email in the future for people that use toolkit to come up with plan that emails them for 3-6 months later to ask about how evaluation went, what they used it for, etc. (2) - Allow people to skip guidance page if not necessary (e.g., returning to site) (1) - Use videos to add guidance on how to use toolkit (1) | - Allow people to have multiple accounts (2) - Save toolkit choices to go back and review plans from previous evaluations (2) - Allow sharing functions so that people can collaborate on one toolkit (2) |
| Measures | - Add a little ‘I’ for information pop-up: definition, amount of questions, time to completion, link to questionnaire (5) - Include cue on how data can be collected below indicator (3) - Define RE-AIM domains (3) - Remove items that look like checkboxes when they function like dropdowns (2) - Include definitions of measures (2) - Include who should collect data (1) - Add command codes for navigating page so someone with a screen reader can navigate (1) - Align pages within build tool when opening and closing tabs (1) - Make sure there are no spelling mistakes or typos (1) | - Include a ‘clear-all’ button (1) - Accessibility adjustments (1)* | Limit to only using categories of RE-AIM that user actually wants to reduce options (1) | - Add suggested algorithms so people know what others have used after they have selected something (1)  (e.g., “other users who have published data also selected these outcomes”) - Add a ‘search’ button (1) - Add filter button with filters such as time to complete, previous uses, etc. (1) |
| Output | - Make sure measures are auto saved or a pop-up that says ‘if you leave this page without saving, data will be lost’ (5) - Make sure that all questionnaire links are valid or use PDF (4) - Have link pop-up in another tab to keep things saved (3) - Make sure it says that link will pop-up in separate page for those with visual impairments (2) - Make sure the entire output can be customized/adapted (e.g., change words if necessary) (2) - Better way to preview specific surveys (1) | - Add additional instructions that this is just an evaluation plan not just a sheet to directly hand out to participants (2) |  |  |

*Note:* Number in parentheses represents the number of individuals who suggested the recommendation. All ‘Must-have’ recommendations were implemented and two ‘Should-have’ recommendations were implemented (see *).
